# Hyperuricemia as an Independent Predictor of Vascular Complications and Mortality in Type 2 Diabetes Patients: A Meta-Analysis

**DOI:** 10.1371/journal.pone.0078206

**Published:** 2013-10-24

**Authors:** Yili Xu, Jiayu Zhu, Li Gao, Yun Liu, Jie Shen, Chong Shen, Glenn Matfin, Xiaohong Wu

**Affiliations:** 1 Department of Endocrinology, the First Affiliated Hospital with Nanjing Medical University, Nanjing, China; 2 Department of Geriatrics, the First Affiliated Hospital with Nanjing Medical University, Nanjing, China; 3 Department of Epidemiology & Biostatistics, School of Public Health, Nanjing Medical University, Nanjing, China; 4 International Diabetes Center, Minneapolis, Minnesota, United States of America; University of Milan, Italy

## Abstract

**Background:**

Recent data have suggested that serum uric acid (SUA) level is positively associated with the development of type 2 diabetes (T2DM). Whether SUA is also independently associated with the development of vascular complications and mortality in T2DM is controversial.

**Methods:**

A computerized literature search of MEDLINE, Embase and PubMed database was conducted and the odds ratio (OR) or hazard ratio (HR) for per 0.1mmol/l increase in SUA in each study was calculated. Cochrane’s Q and I^2^ statistics were used to evaluate heterogeneity among studies and pooling OR and HR with 95% confidence intervals (CIs) were calculated using random-effects models and fixed-effects models. The pooled analysis was performed using Stata 10.0.

**Results:**

Our search yielded 9 eligible articles (16 ORs and HRs) including 20,891 T2DM patients. Pooled estimates for the relationship suggested that each 0.1 mmol/l increase in SUA resulted in a 28% increase in the risk of diabetic vascular complications and a 9% increase in the risk of diabetic mortality. In stratification-analysis, the positive relationship between SUA and vascular complications remained significant irrespective of mean age, adjustment for metabolic variables and medications. However, it was inconsistent in different populations (significantly positive in the Asian but not in Australian and Italian population) and sample sizes (significantly positive in the relatively large sample size [≥1000] but non-significant in the small sample size [<1000]).

**Conclusions:**

Results of this meta-analysis supported elevated SUA as an independent predictor of vascular complications and mortality in T2DM patients. SUA-lowering therapies might be helpful for prevention and treatment of vascular complications in this population.

## Introduction

Type 2 diabetes mellitus (T2DM) is undoubtedly one of the most challenging health problems in the 21st century and the number of diabetic patients diagnosed has reached 366 million in 2011 [1]. Complications due to diabetes are a major cause of disability, reduced quality of life and death. The number of patients diagnosed each year with macrovascular and microvascular complications attributed to T2DM is rising [2,3]. Cardiovascular diseases and nephropathy are the leading causes of death for people with T2DM and type 1 diabetes mellitus, respectively. Therefore, much epidemiologic evidence were committed to risk factors related to development of T2DM and its complications.

Serum uric acid (SUA), the product of purine metabolism, used to be thought predominantly as a predictor of gouty diathesis [4]. However, as a member of metabolic syndrome (MetS), uric acid (UA) could worsen insulin resistance by disturbing insulin-stimulated glucose uptake [5]. And, two recent meta-analysis revealed that elevated SUA has been an independent risk factor for the development of type 2 diabetes (T2DM) [6,7]. Additionally, SUA could also lead to endothelial dysfunction by inhibiting the bioavailability of nitric oxide (NO), the progression of which might cause vascular lesions and even death [8]. Thus, whether SUA is also independently associated with the development of vascular complications and mortality in T2DM is essential for its secondary and tertiary prevention. 

Recently, Kim et al suggested that elevated SUA was associated with diabetic nephropathy[9]. Zoppini et al found that elevated SUA concentrations independently predicted cardiovascular mortality in T2DM [10]. However, Ong et al revealed that SUA did not predict cardiovascular or all-cause mortality in T2DM [11]. Various types of study populations and study designs might have contribution to these disparate findings. Thus, the aim of our meta-analysis was to ascertain the role of elevated SUA in the development of complications and mortality in T2DM derived from previously published studies and to evaluate the effect of study characteristics on this association.

## Materials and Methods

### Study selection

English language publications were identified from Medline (1946.1.1~2012.8.30), Embase (1960.1.1~2012.8.30) and PubMed (1946.1.1~2013.4.9) databases. Search terms and strategies for Medline were as follows: ("Diabetes Mellitus"[Mesh] AND "Uric Acid"[Mesh]) OR ((Diabetes Mellitus OR diabetic) AND Uric Acid).

### Eligibility criteria

The present meta-analysis follows the Preferred Reporting Items for Systematic Review and Meta-analyses (PRISMA) statements (Checklist S1). Articles included should meet our following criteria: 1) one or more type 2 diabetic complications or diabetic mortality as a dominant outcome; 2) measurement of SUA concentration at baseline; 3) OR or relative risk, RR (HR) were provided; 4) in English. Articles were excluded if 1) the outcome was not T2DM; 2) the baseline SUA level was not assessed. 3) OR or RR (HR) were not given. If data of two or more articles were derived from the same subjects, only the most recent one was included in this analysis. Reviews, commentary articles, and editorials were excluded.

### Data extraction and synthesis

Data extracted for this study included the first author’s name, publication year, sample size, duration of follow-up (for cohort studies), population derived from, mean age, gender, adjusted ORs and HRs (95%CI) and multivariable adjustment. If a study provided several RRs (HRs) or ORs, such as unadjusted and adjusted RRs, the most completely adjusted RR (HR) or OR was used. Two of the investigators independently screened and assessed each of the potential titles, abstracts and/or full-texts to determine inclusion. Any disagreement was resolved by a third investigator. We contacted corresponding authors of the original articles to request original patient-level data. 

### Statistics

In order to quantify the dose-response relationship between the baseline SUA level and development of diabetic complications and mortality, we calculated the ORs and HRs for each 0.1mmol/l increase in SUA in each study. For the study that reported [12] by ranges of SUA, we estimated the midpoint in each category by calculating the average of the lower and upper bound. When the highest or the lowest category was open-ended, it was assumed that the open-ended interval length had the same length as the adjacent interval [13]. The variance of the log OR or HR from each study was calculated by converting the 95% confidence interval (CI) to its natural logarithm (width of the CI divided by 3.92). The overall Estimates and its 95% CI could be calculated by exponentiation of the pooled log OR or HR [14]. The Cochrane Q and I^2^ statistics was carried out to assess heterogeneity across studies. For the Q statistic, a P value <0.10 was considered statistically significant for heterogeneity; for I^2^, a value >50% is considered a measure of severe heterogeneity. If test for heterogeneity was not significant, summary estimates of ORs or effect sizes and 95% CIs for the estimates were derived using a fixed-effects model; otherwise, a random-effects model was used [15]. 

Sensitivity analysis was applied to calculate the overall homogeneity and effect size by excluding one study at a time. For instance, we removed the most weighted article from the analysis then continued a meta-analysis with remaining articles. Additionally, stratification-analysis was used to assess a potential difference in distinct population characterized by different features, such as age (≥65y or <65y), population (Italian, Australian and Asian), sample size (≥1000 or <1000) or adjustment for metabolic variables and medications. The adjustment for metabolic variables were regarded as sufficient when the risk estimate was adjusted for more than three factors among obesity, hypertension (or systolic blood pressure), fasting plasma glucose, HDL cholesterol, and triglycerides (or hyperlipidemia or dyslipidemia). Medications indicated drugs usage that could influence SUA concentration, which include allopurinol, ACE/ARB and statin, diuretics usage. Pooled ORs or HRs of complications in T2DM for each 0.1mmol/l increase in SUA within the strata of each study characteristic are indicated.

A funnel plot and Egger’s linear regression test were used to investigate any possible publication bias. All statistical analyses were performed using STATA, version 10.0 (STATA, College Station, TX, USA). A two-tailed P value<0.05 was considered to be significant.

## Results

### Literature search

**Figure 1:**
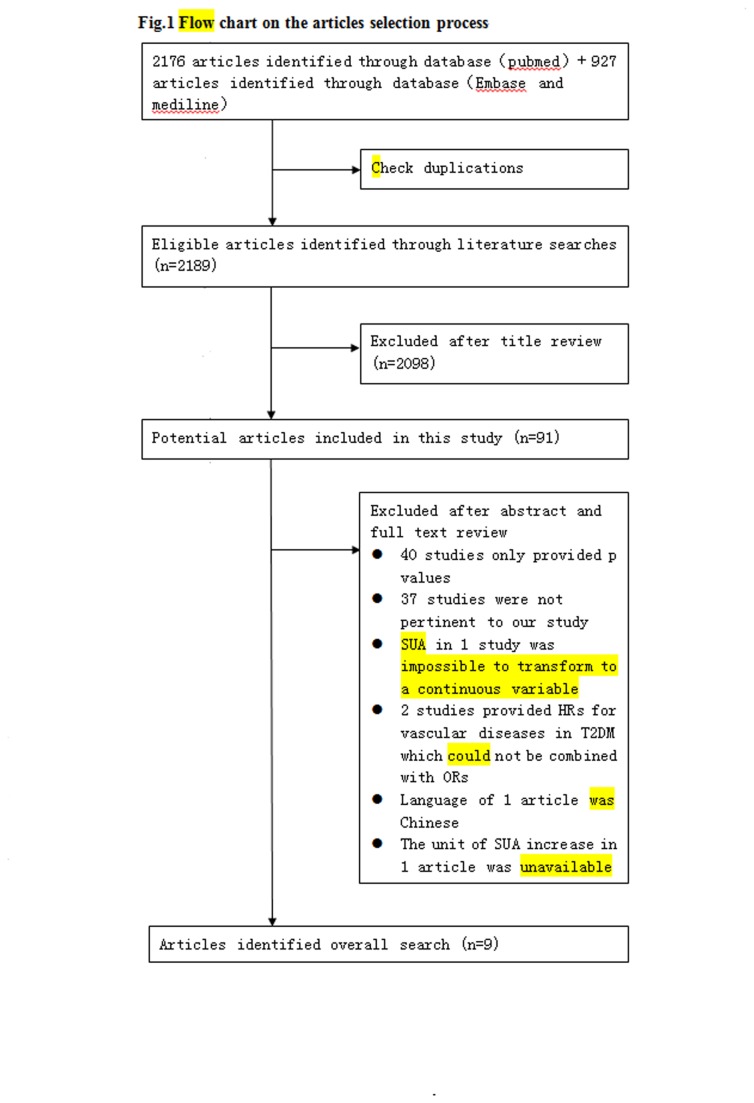
Flow chart on the articles selection process.

Our literature search produced 2189 eligible citations after excluding duplications retrieved from Medline, Embase, and Pubmed databases for the years 1960.1.1~2013.4.9. In total, 91 articles met the criteria after checking the title, 40 studies only providing p values were excluded because the pooling effect of OR was more reliable than that of sum p. Another 37 studies were not pertinent to our study according to the abstract. One study from the remaining 14 articles was excluded for the SUA concentration was impossible to transform to a continuous variable. Of the remaining 13 articles, only one provided HR of the association of SUA and diabetic nephropathy, one provide HR of the association of SUA and diabetic cardiovascular diseases, which cannot be combined with ORs, thus, two of them could not be used in our meta-analysis. Another 2 articles were excluded because one of them was in Chinese and the unit for SUA increase was unavailable in another one. Taken together, reasons for exclusion of 82 articles are listed in Figure 1. 

Nine articles provided ORs and HRs of the relationship between SUA and complications and mortality in T2DM, in which 5 ORs confined to relationship of SUA and macroangiopathy, 7 ORs referred to microangiopathy and 4 HRs pointed to mortality. Ultimately, 9 articles (16 ORs and HRs) including 20,981 participants were included in this meta-analysis [2,9-11,16-20].

### Study characteristics

Characteristics of the 9 articles that provided ORs and HRs on relationship between SUA and complications in patient with T2DM are shown in Table 1. The study design was case-control in 6 studies and follow-up in 3 articles. The studies included publications from 2003 to 2012 and number of subjects ranged from 504 to 11247. Age ranged from 25 to 70. All of the articles included both men and women and percentage of men listed in Table 1. Multivariable adjustments for SUA, age, sex, duration of T2DM, smoking status and risk factors for cardiovascular diseases were considered in most studies, except for 2 studies, in which such data were not available. Five studies considered medications and the correction of MetS was sufficiently considered in 4 studies (Table 1).

**Table 1:** Characteristics of 9 articles (16 ORs and HRs) provided ORs or HRs focusing on relationship of SUA and macrovascular disease, microvascular disease, mortality in T2DM.

Lead author’s name	Publication year	Study design	Duration of follow-up (year)	No of subjects	Study Population	Mean age	Percent of men (%)	complications	Adjusted OR (95%CI)	Multivariable Adjustment
Tapp [2]	2003	cc	/	11 247	Australian	≥25	51.4	PVD	1.03 (1.00-1.06)	NA
								Neuropathy	1.59 (1.21-2.09)	
Tseng [16]	2004	cc	/	508	Chinese	63.8±10.6	41.3	PVD	1.49(1.11-2.22)	age, BMI, duration of diabetes, hypertension and SBP
Cai [17]	2006	cc	/	526	Chinese	55.9	60.2	Retinopathy	1.06(0.98-1.13)	age, sex, duration of diabetes, SBP, HbA1c, CRP, urine albumin, HDL-C, creatinine, BUN
Zoppini [10]	2009	ch	4.7	2726	Italian	67.3±9.6	55.3	CHD-M	1.28(1.01-1.65)	age, sex, BMI, smoking, hypertension, diabetes duration, A1C, dyslipidemia, medication use (allopurinol or hypoglycemic, antihypertensive, lipid-lowering, and antiplatelet drugs), estimated GFR, albuminuria
								All-cause M	1.08(0.96-1.29)	
Ong [11]	2010	ch	/	1,268	Australian	64.1±10.5	48.6	CHD-M	1.06 (0.92-1.22)	demographics, cardiovascular risk factors, cardiovascular/other medications
								All-cause-M	1.05 (0.95-1.17)	
Kim [9]	2011	cc	/	504	Korean	57.3±13.9	47.4	Nephropathy	1.79(1.12-2.86)	age, sex, smoking status, waist circumference, TC, HDL-C, GFR, duration of diabetes, HbA1c, hypertension, use of ACE/ARB and statin, metabolic syndrome
Ito [18]	2011	cc	/	1,213	Japanese	64.0±12.0	59.0	CHD	0.81(0.46-1.41)	age, sex, BMI, duration of diabetes, smoking status, drinking status, treatment for diabetes mellitus, use of diuretics, hyperlipidemia and HbA1c
								PVD	1.72(0.83-2.90)	
								CVD	0.90(0.43-1.79)	
								Retinopathy	1.66(0.99-2.78)	
								Nephropathy	3.40(2.08-5.62)	
								Neuropathy	0.80 (0.44-1.47)	
Giacomo [19]	2012	cc	5.0	1449	Italian	66.1±9.9	61.3	Nephropathy	1.25 (1.04-1.75)	Age, BMI, smoking status, duration of diabetes, systolic blood pressure, and use of antihypertensive drug, insulin therapy, HbA1c, eGFR, albuminuria
Panero[20]	2012	ch	NA	1540	Italian	68.9	NA	All-cause M	1.09 (1.03-1.16)	NA
								CHD-M	1.04(0.95 -1.14)	
								nonCHD-M	1.14(1.04-1.25)	

Cc: case control study; ch: cohort study; CVD: cerebral vascular disease; CHD: Coronary heart disease; PVD: peripheral vascular disease; BMI : body mass index; SBP: systolic blood pressure; HbA1C: glycated hemoglobin A1C; CRP: c-reactive protein; TC :total cholesterol; HDL-C: high-density lipoprotein cholesterol; eGFR: estimated glomerular filtration rate; NA: not available.

### Overall and stratified analysis

**Figure 2:**
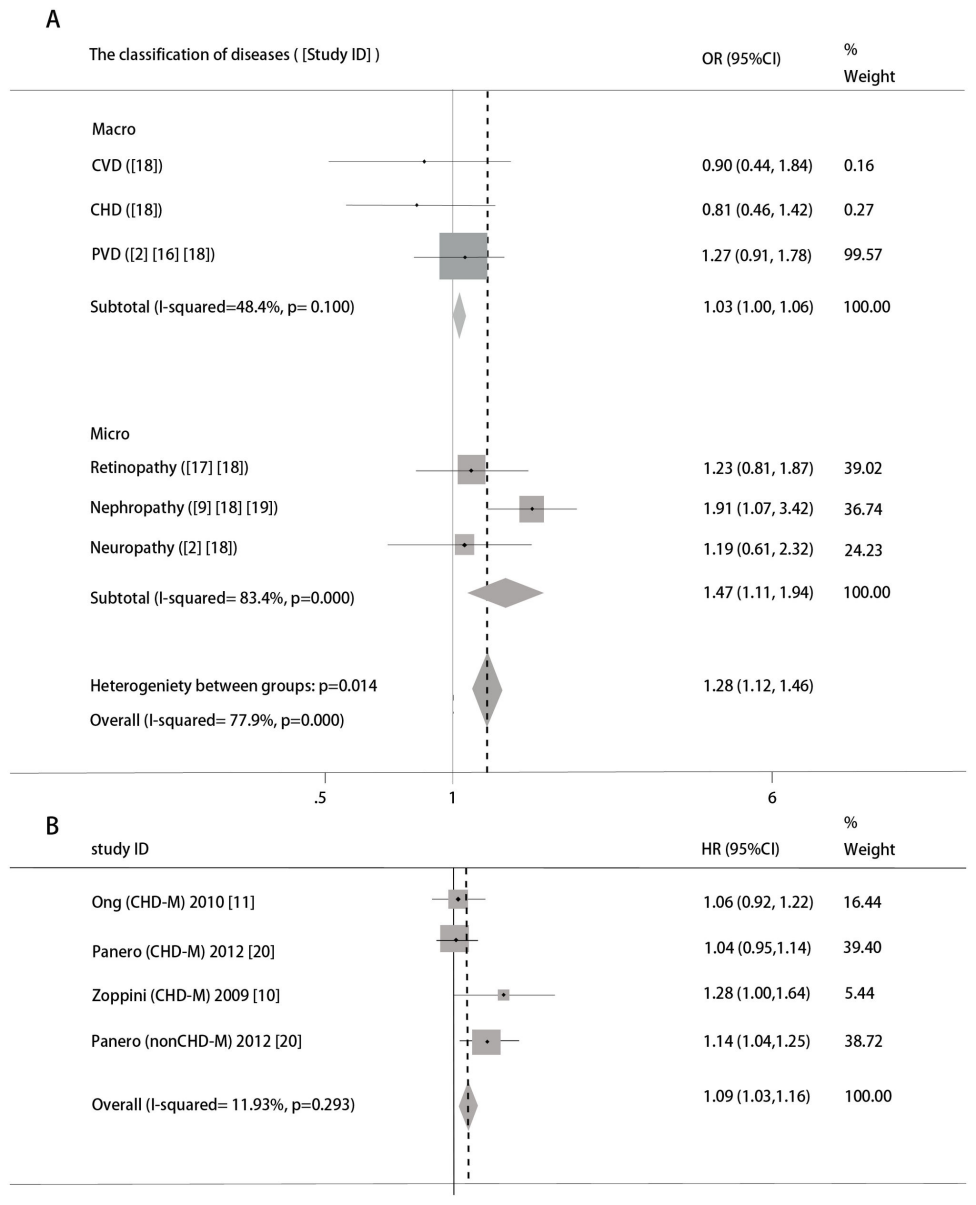
Forest plot of relationship between SUA and vascular complications and mortality in T2DM. The area of each square is proportional to study weight. The diamonds indicate overall estimates (with corresponding 95% CIs). A. Forest plot of relationship between SUA and type 2 diabetic vascular complications. The grey squares and horizontal lines indicate study-specific ORs (with corresponding 95% CIs) for risk of diabetic vascular complications for 0.1mmol/l increase in SUA. The number in the brackets indicated Study ID. B. Forest plot of relationship between SUA and type 2 diabetic mortality. The grey squares and horizontal lines indicate study-specific HRs (with corresponding 95% CIs) for risk of diabetic mortality for 0.1mmol/l increase in SUA. CHD-M and non CHD-M indicated mortality caused by cardiovascular and non-cardiovascular complications. The number in the brackets indicated Study ID.

A forest plot of studies included on the association between elevated SUA and type 2 diabetic complications and mortality was presented. Pooled estimates showed a significantly positive correlation between SUA and type 2 diabetic vascular complications (OR 1.28, 95% CIs 1.12 to 1.46) (Figure 2A), with a statistically significant heterogeneity between studies using the random effects model (P< 0.05, I^2^=77.9%) and a significantly positive relationship of SUA and mortality in T2DM (HR 1.09, 95% CIs 1.03 to 1.16) was observed (Figure 2B) with no evidence of statistical heterogeneity by the method of fixed effects model (p=0.293, I^2^=19.3%). The association was statistically significant both in macrovascular group (the pooled ORs (95% CI) was 1.03 (1.00-1.06)) and microvascular group (1.47 (1.11-1.94)). Interestingly, when vascular complications were classified into each disease, there was a non-significant relationship between SUA and cerebrovascular disease (CVD), coronary heart disease (CHD), peripheral vascular disease (PVD), retinopathy and neuropathy. Furthermore, elevated SUA almost doubled the risk of diabetic nephropathy (DN) (1.91 (1.07-3.42)).

In the sensitivity analysis to evaluate the stability of the relationship between SUA and type 2 diabetic complications and mortality, the significantly positive correlation remained between elevated SUA and vascular complications (1.38 (1.11-1.71)) and mortality (1.12 (1.05-1.22) in T2DM. 

In the stratified-analysis, first, the significantly positive correlation between SUA and type 2 diabetic vascular complications remained in Asian population (1.37 (1.04-1.82)), whereas, not in Italian population (1.25 (0.97-1.62)) and Australian population (1.25 (0.82-1.91)). And a significantly positive result existed in mortality attributed to elevated SUA in Italian (HR [95% CI] equaled to (1.10 (1.03-1.17)) but it was absent in the Australian (1.06 (0.92-1.22)). Second, the significantly positive relationship of SUA and type 2 diabetic vascular complications existed both in the olders (≥ 65y) (1.36 (1.08-1.71)) and the relative younger group (1.25 (1.08-1.45)). Third, the correlation was not significant in the small sample size group (<1000) (1.34 (0.95-1.88)), it was presented in the relatively large sample size stratum (≥1000) (1.32 (1.03-1.70). Additionally, the significantly positive relationship between SUA and vascular complications in T2DM remained after correction for MetS (1.34 (1.03-1.73)) and adjustment for SUA-lowering medications use (1.40 (1.02-1.93)). And, the relationship remained significant in mortality (1.28 (1.00-1.64)) after MetS correction, however, it disappeared after medication adjustment (1.11 (0.98-1.26)). The detail information of stratified analysis was listed in Table 2 and Table 3.

**Table 2:** Stratified analysis to summary odds ratio for the relationship between SUA and T2DM vascular complications.

Subgroup	Number of HRs or ORs	HRs or ORs (95%CI)	Tests for heterogeneity		
			Q	P	I-squared (100%)
Population					
Italian	1	1.25 (0.97-1.62)	0.0	0.0	0.0
Australian	2	1.25(0.82-1.91)	9.59	0.002	89.6
Asian	9	1.37(1.04-1.82)	35.09	0.0	77.2
Age					
<65	4	1.25(1.08-1.45)	42.99	0.001	79.1
≥65	8	1.36(1.08-1.71)	1.73	0.188	42.2
Sample size					
<1000	3	1.34(0.95-1.88)	8.20	0.017	75.6
≥1000	9	1.32(1.03-1.70)	40.69	0.0	80.3
Adjustment					
MetS	9	1.34(1.03-1.73)	33.10	<0.05	75.8
Medications	8	1.40(1.02-1.93)	23.32	0.001	70.0

**Table 3:** Stratified analysis to summary hazards ratio for the relationship between SUA and T2DM mortality.

Subgroup	Number of HRs or ORs	HRs or ORs (95%CI)	Tests for heterogeneity		
			Q	P	I-squared (100%)
Population					
Italian	3	1.10 (1.03-1.17)	3.50	0.174	42.8
Australian	1	1.06 (0.92-1.22)	0.0	0.0	0.0
Adjustment					
MetS	1	1.28 (1.00-1.64)	0.0	0.0	0.0
Medications	2	1.11 (0.98-1.26)	1.70	0.192	41.3

### Assessment of publication bias

The Begg’s funnel plot was presented essentially symmetrical. Little evidence of publication bias was found using Egger’s regression test (P-values for macrovasular,, microvascular complications and mortality were 0.489, 0.068 and 0.490).

## Discussion

This meta-analysis summarizes the available evidence on the relationship between SUA and the development of vascular complications and mortality in T2DM, which provided a meta-analysis of 9 relevant studies - involving a total of more than 20,981 sample size. The overall findings suggested a significantly positive correlation with each 0.1 mmol/l increase in SUA leads to a 28% increase for the risk of vascular complications in T2DM and a 9% increase for the risk of mortality. The relationship between SUA and vascular complications remained significantly positive irrespective of mean age, adjustment for metabolic variables and medications. However, it was inconsistent in different populations (significantly positive in Asian but not in Australian and Italian) and different sample size (significantly positive in relatively large sample size (≥1000) but non-significant in small sample size (<1000)). To our knowledge, the present study is the first study addressing the relationship between SUA and the development of vascular complications and mortality in T2DM.

Vascular lesions were most common complications in T2DM patients. The association between SUA and macrovascular and microvascular complications has not been well established. Results of the relationship of macrovascular complications and SUA in T2DM were conflicting. Several studies supported SUA was an essential predictor for diabetic macrovascular complications including stroke [21], CHD [22] and PVD [16,23]. Nevertheless, the result of a recent publication indicated SUA was not related with CHD in 752 diabetic patients aged 40 years or older using a logistic regression model [24]. In our stratified-analysis, a significantly positive relationship presented in the strata analysis of macrovascular group (the pooled OR (95% CI) is 1.03 (1.00-1.06)). 

Our stratified-analysis indicated elevated SUA provides higher microvascular risk (OR (95%CI) = 1.47 (1.11-1.94)). As regards to DN, epidemiological research showed consistent results of a significant correlation between SUA concentration and nephropathy in T2DM [18,19,22,25]. In our stratified-analysis a significantly positive correlation also confirmed (OR (95%CI) = 1.91 (1.07-3.42)). As regards to retinopathy, it has been established that SUA increase positively related with the progression of retinopathy in cross-sectional studies [26,27]. However, after using logistic regression analysis, Cai et al. showed that SUA wasn’t independent risk factor of diabetic retinopathy in elderly T2DM patients [17]. Furthermore, Felderman et al. followed 95 consecutive diabetes clinic patients for 15 years, which indicated higher SUA levels were not related to diabetic retinopathy in a prospective study [28]. Overall, in the strata analysis of retinopathy in our study, no statistical significance was present (OR (95%CI) = 1.23 (0.81-1.87)). As for the neuropathy, studies presented controversial results [2,18]. A recent study documented a significantly positive association between higher SUA concentration and neuropathy (P<0.001) [29]. However, our neuropathy stratum (OR (95%CI) = 1.19 (0.61-2.32)) indicated a non-significant relationship, which could be partially due to limited number of studies or relatively short duration of follow-up.

Previous studies have indicated that elevated SUA level increases the risk of all-cause and CHD-mortality dealing with T2DM alone or mixed with the general population [30,31]. On the contrary, Ong et al. recently reported that the SUA level did not predict cardiovascular mortality in 1,268 diabetes patients in Australia [11]. Furthermore, a J-shaped manner of association between SUA and CHD mortality was presented in elderly subjects with diabetes [32]. Even results were remained unclear, our stratified-analysis indicated a significantly positive relationship between SUA and type 2 diabetes mortality (OR (95%CI) = 1.09 (1.03-1.16)).

Taken together, is it possible to establish whether the observed significantly positive relationship for vascular complications is causal or non-causal or both? Take DN as an example, besides epidemiology results discussed above, firstly, DN might lead to SUA increase by reducing renal UA secretion [33-35] which could confound the relationship. Secondly, uric-acid-influencing medication use during DN therapy could be one of the confounders for their relationship. Thirdly, insulin resistance, one of the markers of MetS was observed to be associated with both HUA [36] and DN [37]. Thus, we could not exclude the possibility that SUA and DN were non-casually related. However, there were analogies which indicated a possibility of a causal association. First of all, a meta-analysis [38] combined 21 eligible cohort studies with large sample size (n=279,805) suggested SUA is also an independent risk factor for incidence of kidney diseases in general population. And then, one of included studies suggested a significantly positive association in the stratum adjusted for sufficient metabolic confounders (≥3 metabolic components) and drugs influencing SUA levels [9]. Finally, there were also several intervention findings for DN both in animals [39] and humans [40] suggesting beneficial effects of reducing SUA on diabetic nephropathy, which could contribute to the evidence for the role of SUA and DN. Furthermore, the significantly positive relationship was confirmed in our stratified-analysis, which suggested that the risk of DN for each increase of SUA was nearly doubled (1.91 (1.07-3.42)). Thus, HUA might increase the risk of DN. Moreover, the significantly positive association between SUA and type 2 diabetes complications is biologically plausible. Elevated SUA causes endothelial dysfunction by inhibiting NO production and preventing reactive oxygen species formation [41], overproduction of mitochondrial Na(+)/Ca(2+) exchanger-mediated mitochondrial calcium [42], activating renin–angiotensin system [43], causing proinflammation by stimulating monocyte chemoattractant protein-1 [44], vascular smooth muscle cell proliferation [45], platelet aggregation [46], and endothelin increase [47], which eventually contributing to both vascular complications. Therefore, HUA might increase the risk of DN while DN accelerates the elevation of SUA. As to macrovascular complications, novel findings suggested that lowering SUA may also help to reduce the risk of cardiovascular diseases [48]. As regards to mortality, previous studies indicated conflicting results about the effect of lowering uric acid on mortality [49-51]. Pooled estimate in our study indicated a 9% risk for mortality of per 0.1mmol/L of SUA, thus, even the positively significant relationship of SUA and mortality was observed only in Italian in our stratified-analysis, the non-significant results discovered in Australian population might be due to limited sample size. Overall, it was still too early to tell whether the relationship between SUA and diabetic complications and mortality is causal or accompanying. Thus, further studies on the effects of SUA-lowering-therapy were warranted for vascular complications and mortality in T2DM. 

The limitations of this meta-analysis need to be considered. Firstly, the OR and HR calculation for per 0.1mmol/l increase in SUA in order to quantify the dose-response relationship between the baseline SUA level and incidence of diabetic vascular complications may overestimate the magnitude of any publication bias [6]. And, we cannot determine that the role of SUA on the risk of vascular complications and mortality in T2DM is a dose response or a threshold effect. Secondly, significant heterogeneity was observed, thus random effect models were used which could provide studies with small sample size a higher weight to decrease the influence of heterogeneity and subgroup analysis were conducted to investigate the source of heterogeneity. Furthermore, sensitivity-analysis was conducted to evaluate the stability of our results, and the results remained significant. Thirdly, factors of MetS (including hyperinsulinemia and blood glucose status at baseline) and medications of DN such as angiotensin converting enzyme inhibitor/angiotensin II receptor blocker and statin might confound the relationship between SUA and diabetic vascular complications and mortality. However, Kim et al [9] and Ito et al [18] took consideration of both MetS and drugs, the result of which indicated that elevated SUA was a predictor of DN. 

In conclusion, this meta-analysis indicated that SUA is independently associated with development of diabetic vascular complications and mortality. Further studies should attempt to determine if there is a threshold of SUA level for increased risk of type 2 diabetes vascular complications and mortality. More evidence from epidemiological studies, mechanistic and especially genetics, and large randomized controlled trials especially of SUA-lowering-therapy are warranted.

## Supporting Information

Checklist S1 **PRISMA checklist.**
(DOC)
